# ESR Statement on the Validation of Imaging Biomarkers

**DOI:** 10.1186/s13244-020-00872-9

**Published:** 2020-06-04

**Authors:** European Society of Radiology (ESR), Angel Alberich-Bayarri, Steven Sourbron, Xavier Golay, Nandita deSouza, Marion Smits, Aad van der Lugt, Ronald Boellard

**Affiliations:** Vienna, Austria

**Keywords:** Imaging biomarkers, Metrology, Validation, Accuracy, Precision

## Abstract

Medical imaging capable of generating imaging biomarkers, specifically radiology and nuclear medicine image acquisition and analysis processes, differs from frequently used comparators like blood or urine biomarkers. This difference arises from the sample acquisition methodology. While different analysis methodologies and equipment provide slightly different results in any analytical domain, unlike blood or urine analysis where the samples are obtained by simple extraction or excretion, in radiology the acquisition of the sample is heterogeneous by design, since complex equipment from different vendors is used. Therefore, with this additional degree of freedom in medical imaging, there is still risk of persistent heterogeneity of image quality through time, due to different technological implementations across vendors and protocols used in different centres. Quantitative imaging biomarkers have yet to demonstrate an impact on clinical practice due to this lack of comprehensive standardisation in terms of technical aspects of image acquisition, analysis algorithms, processes and clinical validation.

The aim is establishing a standard methodology based on metrology for the validation of image acquisition and analysis methods used in the extraction of biomarkers and radiomics data. The appropriate implementation of the guidelines herein proposed by radiology departments, research institutes and industry will allow for a significant reduction in inter-vendor & inter-centre variability in imaging biomarkers and determine the measurement error obtained, enabling them to be used in imaging-based criteria for diagnosis, prognosis or treatment response, ultimately improving clinical workflows and patient care. The validation of developed analytical methods must be based on a technical performance validation and clinical validation.

## Key points

- Unlike blood or urine analysis where the samples are obtained by simple extraction or excretion, in radiology the acquisition of the sample (the images) is heterogeneous by design, since complex equipment from different vendors is used.
- The validation of developed analytical methods must be based on a technical performance validation (precision - repeatability and reproducibility - and accuracy assessment) and clinical validation.
- Metrology is the scientific domain in which imaging biomarkers must be validated, as with other measurement devices.

## Patient summary

In a number of areas of medicine, tests such as blood or urine sampling can measure certain characteristics by which a particular disease or biological process can be identified – these characteristics specific to a disease or biological process are called biomarkers. In medical imaging, it is difficult to produce reliable biomarkers because of complexities involved in collecting and analysing data from images. Equipment (scanners) from different manufacturers or the use of different methodologies for data analysis means that the measurements from different institutions or even within the same institution are often difficult to compare. Hence, despite technological advances, his lack of standardisation has prevented imaging biomarkers from significantly impacting clinical practice. This paper discusses how imaging biomarkers can be clinically validated based on metrology - the science of measurement – to ensure that variability between centres is minimised. This should enable imaging biomarkers to be used for diagnosis, prognosis or treatment response, with the ultimate aim of improving clinical workflows and patient care.

## Introduction

Quantitative imaging biomarkers have yet to demonstrate an impact on clinical practice due to the lack of comprehensive standardisation in terms of technical aspects of image acquisition, analysis algorithms, processes, and clinical validation. The development of new imaging biomarkers involves well-defined consecutive steps including proof of concept and mechanism, optimised imaging acquisition protocols, source images, analysis methodology and algorithms, statistical measurements and structured reports [1, 2]. In order to have an impact and improve medical imaging workflow, imaging biomarkers have to be technically and clinically validated and provide additional value and guide radiologists in the diagnosis and assessment process. In order to enable the widespread use of quantitative imaging biomarkers in both clinical and research settings, a full validation methodology has to be established, including a robust technical and clinical validation. A consensus statement seems the best practice to achieve success. This paper aims to establish a standard methodology based on metrology for the validation of image acquisition and analysis methods used in the extraction of biomarkers and radiomics data [3]. The appropriate implementation of the guidelines herein proposed by radiology departments, research institutes and industry will allow for a significant reduction in inter-vendor & inter-centre variability in imaging biomarkers and determine the measurement error obtained, enabling them to be used in imaging-based criteria for diagnosis, prognosis or treatment response, ultimately improving clinical workflows and patient care. The validation of developed analytical methods must be based on a technical performance validation (precision and accuracy assessment) and clinical validation.

Medical imaging capable of generating imaging biomarkers, specifically radiology and nuclear medicine image acquisition and analysis processes, is fundamentally different from frequently used comparators like blood or urine biomarkers. This difference arises from the methodology for obtaining the sample. While different analysis methodologies and equipment provide slightly different results in any analytical domain, unlike blood or urine analysis where the samples are obtained by simple extraction or excretion, in radiology the acquisition of the sample (the images) is heterogeneous by design, since complex equipment from different vendors is used. Therefore, with this additional degree of freedom in medical imaging, there is still a risk of persistent heterogeneity of image quality through time, due to different technological implementations across vendors and protocols used in different centres. Standardization of image quality as an input for different imaging biomarkers analysis will never be fully achieved and there is a certain risk of reaching an operational limit. This risk should be identified early enough to enable corrective measures, for instance by exploring new ways to change the heterogeneity trend by the use e.g. of artificial intelligence (AI) based approaches, in order to let complex and deep neural networks learn from the lack of homogeneity in the collected images, both in the DICOM metadata and in the pixel information.

The Quantitative Imaging Biomarkers Alliance (QIBA) initiative from the Radiological Society of North America (RSNA) has proposed an imaging biomarkers qualification profile that is being adopted among different types of imaging measurements procedures [4]. In particular, these so-called Imaging Biomarker Profiles set the ground rules for achieving the best possible standardisation at the acquisition level, as well as the minimum requirements for the image analysis software used. These Profiles end with the technical and clinical validation of biomarkers, although so far, no biomarker has yet reached the final milestone. This paper aims to provide an overview of the main technical and clinical validation steps general to all biomarkers, based on metrological principles.

### Technical validation

**Fig. 1:**
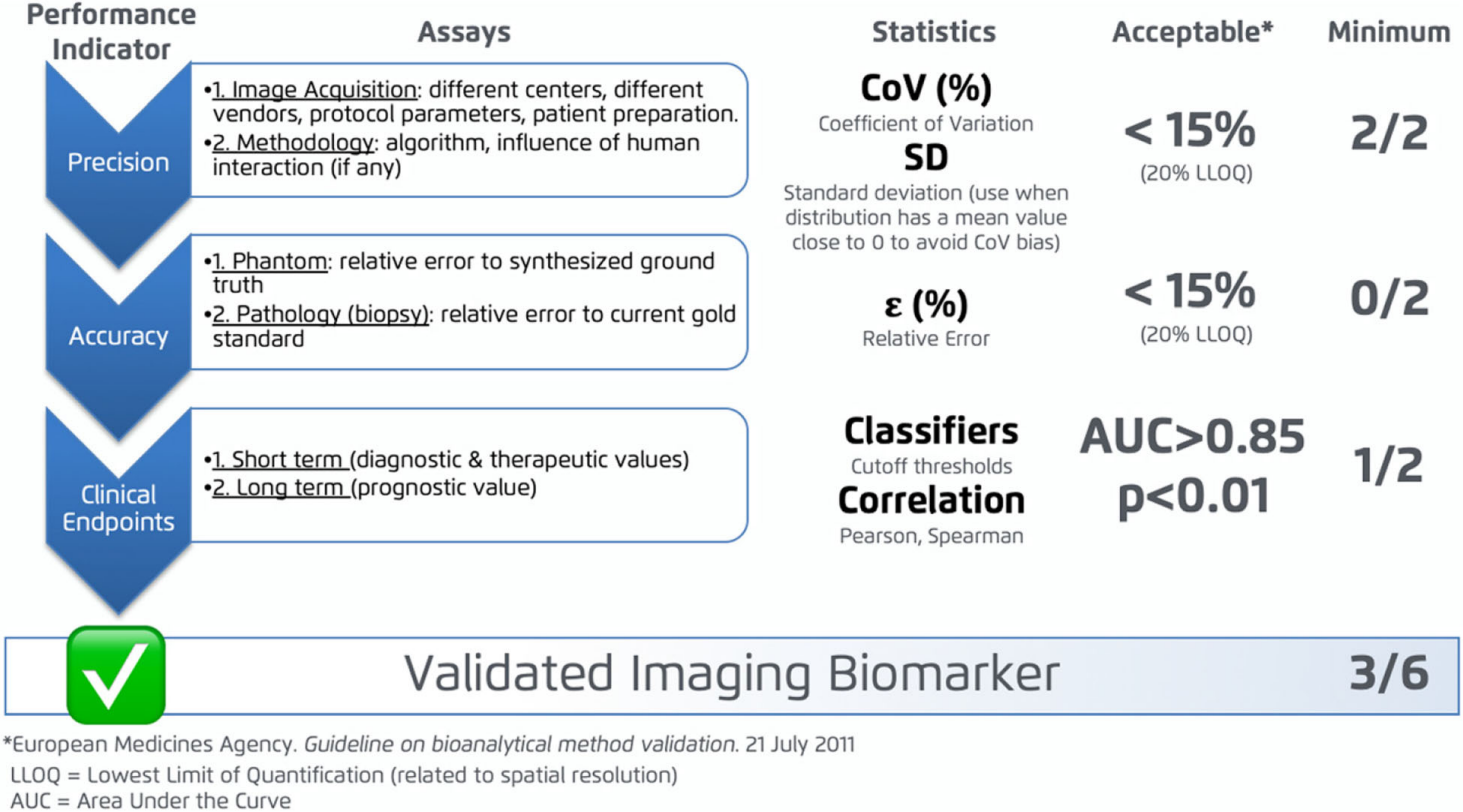
Imaging Biomarkers Validation Pipeline (adapted from [6])

Although there is no current consensus on how to validate imaging biomarkers, a standardised validation profile should comprise three steps (see Fig. 1), in which the main factors influencing and introducing uncertainty in measurements have been considered. In order to be aligned with already existing bioanalytical validation strategies, including the QIBA guidelines, the pipeline proposed for technical validation follows the guidelines for the evaluation of bioanalytical methods from the European Medicines Agency (EMA) [5]. The EIBALL profile herein introduced establishes that the biomarkers need to be validated in terms of their Precision, Accuracy and Clinical Relationship.

Metrology is “the science of measurement, embracing both experimental and theoretical determinations at any level of uncertainty in any field of science and technology”, as defined by the International Bureau of Weights and Measures (BIPM). Therefore metrology is the appropriate scientific domain within which imaging biomarkers must be validated, as for any other measurement devices. The technical validation of the imaging biomarkers will determine both the Precision and Accuracy of the measurements. According to ISO norm BS ISO 5725-1 (“Accuracy, trueness and precision of measurement methods and results – Part 1: General principles and definitions”), Precision is defined as the closeness of agreement among a set of results, while Accuracy is defined as the closeness of a measurement to the true value.

Unlike Accuracy, Precision can be evaluated for all imaging biomarkers. Precision comprises the characterization of both repeatability and reproducibility. Obtaining a high precision (low variability) is considered mandatory for the validation of an imaging biomarker. For precision evaluation, the Coefficients of Variation (CoV) of the biomarker, the standard deviation (SD), and the inter-quartile ratio (IQR) obtained repeatedly with the variation of different factors, are calculated. The variable factors can be related either to the image acquisition protocol or to the image analysis methodology (both image preparation and application of the quantification algorithms). For the study of repeatability, the methodology is tested and re-tested with the same image acquisition characteristics (i.e. same machine, same protocol). To analyse the influence of the image acquisition on the measurement’s reproducibility however (as opposed to repeatability), the imaging biomarkers should ideally be calculated for the same subjects testing the following main varying conditions by acquiring images:

- in different centres (i.e. images acquired with same protocol in different hospitals)
- using different equipment (i.e. MR images from 1.5 T or 3 T)
- using multiple vendors (i.e. CT images from vendor A, B or C)
- applying different acquisition protocol configurations (i.e. images acquired with different slice thickness and / or kV values)
- performing various patient preparations (i.e. use of anti-peristaltic drugs in quantitative analysis of bowel diffusion by MR)
- using different contrast agents (e.g. from different vendors or with different molecular weights).

For the evaluation of the influence of analysis algorithms on the obtained measurements, imaging biomarkers should be calculated for the same subjects and acquisition protocols while testing the following varying conditions:

- Operator influence (intra-operator variability, inter-operator variability)
- Processing algorithm variability (i.e. in case of iterative algorithms that may converge at different steps)

The global CoV should be below 15%, with the exception of measurements below the Lowest Limit of Quantification (LLOQ), in which case the CoV values of the biomarker can be up to 20%. Nevertheless, CoV is known to present important limitations to evaluate measurement variability when the mean of the samples approaches zero (CoV tends to infinity). In such cases, evaluation of the SD is preferred.

Obtaining high precision in the quantitative imaging biomarkers extracted both in radiology and nuclear medicine modalities is critically necessary for their use in multi-center research projects and clinical trials [7].

The Accuracy of the method can be evaluated by comparing the obtained results with a ground truth. The ground truth can be based on information extracted from a tissue sample or from synthetic phantoms (physical or digital reference objects) with different compounds and known properties that emulate the characteristics of the biological tissue. For Accuracy evaluation, the relative error of the imaging biomarker compared to the ground truth must be calculated. The relative error should be below 15%, with the exception of measurements below the LLOQ, in which case the relative error can be up to 20%.

In some cases, there is no reference value available, e.g. because the synthesis of a stable phantom is a complex process, or because the considered reference value also has a high variability and derives from a coarser category-based analysis rather than the continuous domain of imaging biomarkers **(**e.g. steatosis grades in pathology vs. proton density fat fraction quantification from MRI). The lack of ability to calculate Accuracy can be compensated by replacing it with the clinical sensitivity and specificity of the imaging biomarker calculated (i.e.: it is unknown how accurate the method is, but we know that the specific imaging biomarker is significantly related to important disease hallmarks). Note, however, that in that case, the principles leading to the validation of the biomarkers are more related to its clinical usability than its metrological precision, and therefore other principles altogether apply.

### Clinical validation

The main purpose of clinical validation is to show the relationship between the extracted imaging biomarker and the disease status. Not only do imaging biomarkers need to be objective and reproducible, they also have to show a clear efficacy in the detection and diagnosis of disease or in the evaluation of treatment response. The imaging biomarker can be evaluated either as a short-term (assessing detection, diagnosis and evaluation of treatment response) or long term (prognostic patient status) parameter. This diagnostic efficacy must be confirmed by a clear relationship with expected clinically meaningful endpoints, acting as surrogate indicators of relevant clinical outcomes like treatment response prediction, progression-free survival, overall survival, among others. The type and degree of relationship between the imaging biomarkers and clinical variables will be analysed through sensitivity, specificity, statistical difference between clinical groups and correlation studies. Finally, to achieve clinical integration and expand its utility, the methodology must be widely clinically acceptable, easy to implement and cost-efficient.

## Conclusion

The continuous technological advancement and improvements in medical imaging hardware and software require constant reassessment of the quantitative accuracy of evaluation of medical images, radiomic features and regular updates of the standardisation requirements. Imaging biomarkers need to be treated similarly to any validated laboratory test.

## Data Availability

All data generated or analysed during this study are included in this published article.
